# PB.42. Influences upon presentation to the symptomatic breast clinic: is there an increase in symptomatic referrals due to the physical presence of the breast screening van within an area?

**DOI:** 10.1186/bcr3708

**Published:** 2014-11-03

**Authors:** F Wotton, R Green

**Affiliations:** 1 Peninsula Radiology Academy, Plymouth, UK; 2 South Devon Healthcare NHS Foundation Trust, Torquay, UK

## Introduction

Current published literature on health-seeking behaviour in women with a self-detected breast lesion mainly explores the reasons surrounding why a delayed presentation may occur. However, there is no current literature on whether the presence of the National Breast Screening van has any influence on symptomatic referrals. Therefore, the authors propose there will be an increase in symptomatic referrals within the cohort of patients who share a postcode with the breast screening van location.

## Methods

The National Breast Screening van was parked in the TQ4 postcode between 30 November 2012 and 30 June 2013. All new GP symptomatic breast referrals between the above dates and those during the same timeframe of the previous year were recorded by age and postcode. The referral patterns, ages and number of new symptomatic breast cancer diagnoses between were compared between the study timeframes.

## Results

There was a 12% increase in overall referrals during the study timeframes in 2011/12 to 2012/13. There were no significant differences in referral patterns by postcode. However, there was a statistically significant increase in referrals in younger patients with a consequent reduction in those of screening age (*P *< 0.001), when compared with a separate postcode. No difference in new symptomatic breast cancer diagnoses was seen during the two timeframes.

## Conclusion

Whilst the presence of the screening van did not influence overall referrals, there was a shift in age distribution with a statistically significant increase in younger, prescreening women and a consequent decrease in older, screening-age women. The screening van did not influence the number of new symptomatic breast cancers.

